# Repeatability, reproducibility, and agreement of three tonometers for measuring intraocular pressure in rabbits

**DOI:** 10.1038/s41598-021-98762-7

**Published:** 2021-09-28

**Authors:** Christian J. F. Bertens, Ralph J. S. van Mechelen, Tos T. J. M. Berendschot, Marlies Gijs, Jarno E. J. Wolters, Theo G. M. F. Gorgels, Rudy M. M. A. Nuijts, Henny J. M. Beckers

**Affiliations:** 1 Department of Ophthalmology, University Eye Clinic Maastricht, School for Mental Health and Neuroscience (MHeNs), Maastricht University Medical Center+ (MUMC+), P. Debyelaan 25, PO Box 5800, 6229 HX Maastricht, The Netherlands; 2 Chemelot Institute for Science and Technology (InSciTe), Gaetano Martinolaan 63-65, 6229 GS Maastricht, The Netherlands

**Keywords:** Medical research, Sensors and probes, Visual system

## Abstract

The aim of this study was to evaluate repeatability, reproducibility, and agreement of three commonly used tonometers in animal research (TonoLab, TonoVet, and TonoPEN AVIA) in a cohort of 24 rabbits. Additionally, the impact of sedation on IOP was investigated in 21 New Zealand White rabbits with the TonoVet tonometer. Repeatability was determined using the coefficient of variation (CoV) for two observers. For the TonoLab (6.55%) and TonoVet (6.38%) the CoV was lower than for the TonoPEN AVIA (10.88%). The reproducibility was highest for the TonoVet (0.2 ± 3.3 mmHg), followed by the TonoLab (0 ± 12.89 mmHg) and lowest for the TonoPEN AVIA (− 1.48 ± 10.3 mmHg). The TonoLab and TonoVet showed the highest agreement (r = 0.85, R^2^ = 0.73). After sedation, a significant IOP reduction (often > 25%) was observed. Our results show that among the three tonometers tested, the TonoVet tonometer is best for use in rabbits while the TonoLab should be avoided. The impact of sedation on IOP was substantial and should be taken into account during experimentation.

## Introduction

Increased intraocular pressure (IOP) and fluctuations in IOP are important characteristics of glaucoma. Repeatable and reproducible objective measurement of IOP are of great importance for disease management. Animal models are routinely used to study underlying pathophysiology and are used in the development of new glaucoma therapies. For example, in glaucoma animal models the anterior chamber of the eye can be injected with microbeads to block the outflow of aqueous humor (AH), thus increasing IOP^1–3^.

IOP in animal experiments can be measured by manometry or tonometry. Although manometry is the most accurate method, it is invasive and requires trained personnel along with expensive and specialized equipment. Tonometry is an indirect non-invasive measuring method that can be divided into three different subcategories: indentation, applanation, and rebound tonometry. Indentation (also known as impression) tonometry uses a plunger to measure the depth of corneal indentation, as used in the Schiøtz tonometer^4^. In applanation tonometry, the force needed to flatten the cornea is used to calculate IOP. This method is routinely used in regular clinical care, where Goldmann applanation tonometry (GAT) is the gold standard^5,6^. Rebound tonometry determines the IOP via induction of a current generated by the rebound effect of a small probe onto the cornea. The use of rebound tonometry (e.g. iCare tonometers) is gaining popularity in the clinic, especially for children and non-cooperative patients, as the tonometers are handheld devices and no topical anesthesia is required for the procedure^7^.

In animal research, the most commonly used tonometers are the TonoLab (intended for mice and rats), TonoVet (intended for dogs, cats, and horses) and TonoPEN (intended for all animals) (Table 1). Although none of these tonometers have been specifically designed for rabbits and there may be substantial differences between the devices, they are commonly used on rabbits for research purposes.

**Table 1:** Literature overview of the use of different tonometers in animal research.

Animals	ICare TonoLab	ICare TonoVet (Plus)	Reichert TonoPEN (XL, VET, or AVIA)
Frogs	Determination of reference IOP^23,24^	Determination of reference IOP^23–25^	Determination of reference IOP^25^
Turtles	Tonometer validation^26^	Tonometer validation^26,27^	
Mice	Effect of general anesthesia on IOP^28^ Tonometer validation^29,30^ Glaucoma research^31,32^ Effect of CCT on IOP^33^		Tonometer validation^30,34^ Glaucoma research^31^
Chinchillas	Tonometer validation^35^	Tonometer validation^35^ Determination of reference IOP^36^	Tonometer validation^35^
Rats	Tonometer validation^29,30,37–39^ Effect of general anesthesia on IOP^37^		Tonometer validation^30,34,39–41^ Effect of general anesthetics on IOP^42,43^ Circadian variation^44^
Guinea pigs			Determination of reference IOP^45^ Effect of topical drugs on IOP^46^
Ferrets			Determination of reference IOP^47^
Hedgehogs		Determine prevalence of ocular diseases^48^	Determine reference IOP^49^
Rabbits		Tonometer validation^50–54^ Effect of topical drugs on IOP^55^	Tonometer validation^6,50,51,53,54,56^ Effect of topical drugs on IOP^46,57–59^
Dogs		Effect of CCT on IOP^60^ Tonometer validation^61–63^ Glaucoma research^64,65^	Effect of CCT on IOP^60^ Tonometer validation^61,62^ Glaucoma research^64,65^ Effect of topical drugs on IOP^66^ Effect of age on the IOP^67^
Cats		Glaucoma research^65,68^ Tonometer validation^69^ Effect of general anesthetics on IOP^70^ Effect of topical drugs on IOP^55^	Glaucoma research^65^ Tonometer validation^69^
Birds		Tonometer validation^71,72^ Determination of reference IOP^73–77^	Tonometer validation^71^ Determination of reference IOP^73,74^
Cows		Determination of reference IOP^78^	
Horses and donkeys		Determination of reference IOP^79^ Effect of endurance training on IOP^80^	Determination of reference IOP^79^
Pigs		Tonometer validation^81^	Tonometer validation^81^
Alpaca’s		Determination of reference IOP^82^	Determination of reference IOP^82^
Goats and sheep		Determination of reference IOP^83^	
Non-human primates		Tonometer validation^84,85^ Determination of reference IOP^86^	Tonometer validation^85^ Determination of reference IOP^86^ Effect of general anesthetics on IOP^87^ Glaucoma research^88^

CCT, central corneal thickness; IOP, intraocular pressure.

Therefore, we wanted to investigate which tonometer is most suitable for research with rabbits. The TonoLab and TonoVet are both rebound tonometers, whereas the TonoPEN is an applanation tonometer. However, it is unknown which tonometer has the best repeatability and reproducibility when used by multiple observers. Biomechanical factors may also affect IOP readings, such as corneal thickness and stiffness^8–10^, mental stress^11–14^, circadian rhythm^15,16^, and the type of (e.g. general) anesthesia or sedation^17–21^. The use of sedatives is common practice in animal studies and clinical procedures^22^, but the effect of injectable sedatives on IOP has not been fully characterized. Furthermore, different absolute IOP values are commonly observed when various tonometers are compared. Hence, the aim of this study was to compare the repeatability, reproducibility and agreement of the TonoLab, TonoVet and TonoPEN AVIA tonometers, together with investigating the effect of the injectable sedative medetomidine (an α2 adrenergic agonist) on rabbit IOP.

## Materials and methods

### Animals and animal care

Animal procedures were conducted according to the Association for Research in Vision and Ophthalmology (ARVO) Statement for the Use of Animals in Ophthalmic and Visual Research, the Animal Research: Reporting of In Vivo Experiments (ARRIVE) 2.0 guidelines^89^, and the Guidelines of the Central Laboratory Animal Facility of Maastricht University. All protocols were approved by the Central Authority for Scientific Procedures on Animals (CCD, Den Haag, NL) and were in accordance with the European Guidelines (2010/63/EU) (Approved Dutch license number: AVD107002017829 and AVD1070020197464).

New Zealand White (NZW) rabbits (2.0 kg–2.5 kg, males and females) (Envigo (Horst, NL and Bicester, UK) and Charles River (Ecully, FR)) were group housed (maximum 7 animals per cage, males and females separated), and maintained under controlled conditions of temperature and humidity on a 12 h:12 h light–dark cycle. The rabbits had ad libitum access to water and 100 g dried chow per animal and all had a two-week acclimatization period before the start of the experiments. All rabbits were normotensive. At the end of the experiment the rabbits were euthanized with an overdose of pentobarbital sodium, 200 mg/kg (Euthasol 20, Produlab Pharma B.V., NL), intravenously injected.

### Animals

24 NZW rabbits were used (12 males) for the IOP part of the study (AVD107002017829), whereas 21 NZW rabbits (10 males) were used for the sedation part (AVD1070020197464). The rabbits were trained for one week to get used to the restrainer and the IOP measurements. At the start of each experiment, the rabbits were intra muscularly (IM) sedated using medetomidine (0.5 mg/kg) (Sedator, A.S.T. Farma B.V., Oudewater, NL). Prior to the TonoPEN AVIA measurement, the eye was topically anesthetized with 0.4% Oxybuprocaine hydrochloride solution (MINIMS, Bausch & Lomb Pharma, Brussels, BE). After the measurements, the animals were recovered using 1 mg/kg i.m. atipamezole (Antisedan, ORION pharma, Mechelen, BE). For both studies, the left eye of the rabbits was used.

### Tonometers

**Figure 1:**
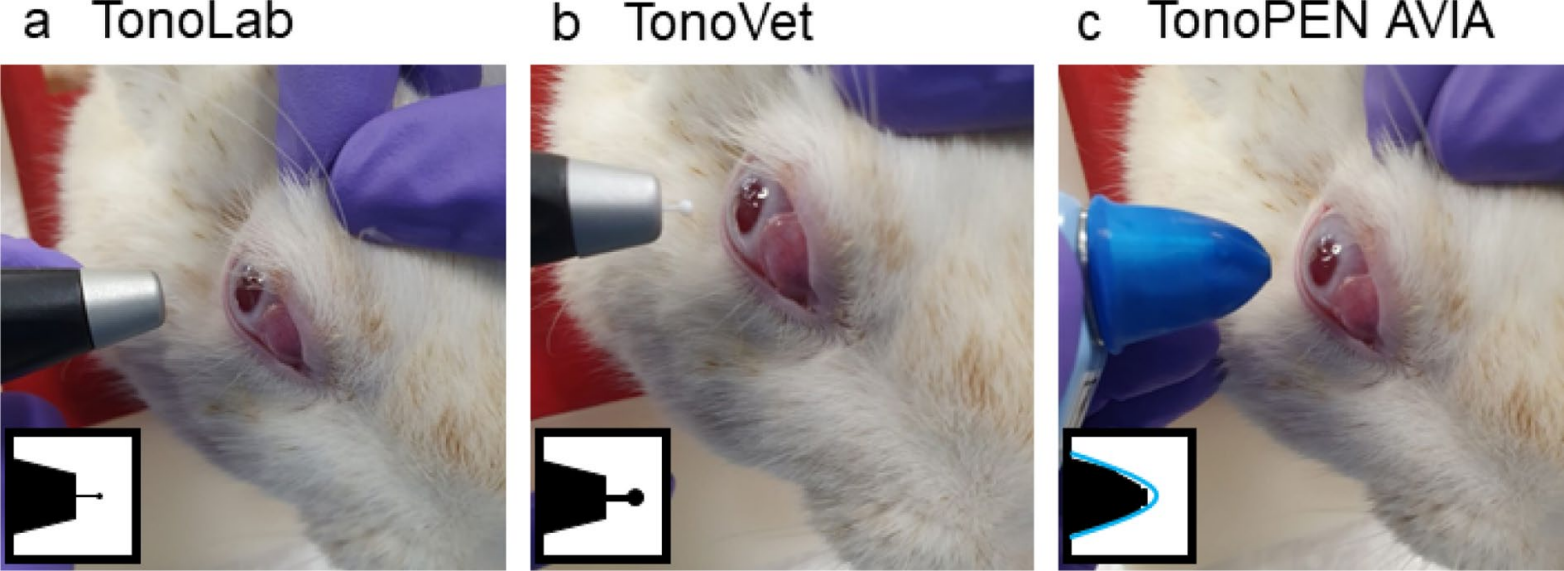
Measuring IOP in a rabbit using different tonometers. (**a**) TonoLab (rebound tonometer), (**b**) TonoVet (rebound tonometer), (**c**) TonoPEN AVIA with a single-use blue Ocu-Film tip-cover (applanation tonometer).

IOP was measured using the iCare TonoLab (iCare Finland Oy, Vantaa, FI) (in rat setting) (Fig. 1a), followed by the iCare TonoVet (iCare Finland Oy, Vantaa, FI) (in dog/cat setting) (Fig. 1b) and finally, Reichert TonoPEN AVIA (AMETEK Inc., Unterschleißheim, DE). An Ocu-Film tip-cover was used for the TonoPEN AVIA (Fig. 1c). The TonoPEN AVIA was used last, due to the potential effect of topical anesthesia on the TonoVET and TonoLab^90^.

### Repeatability, reproducibility, and agreement of three tonometers

To investigate the repeatability of IOP measurements, defined as the ability of the observer to produce similar results time after time, all measurements were performed in triplicate^91^. The average of six readings is reported by the tonometer. According to the manufacturer’s instructions, IOP measurements with a repetition deviation ≥ 1.0 mmHg (TonoLab and TonoVet) or a repetition confidence lower than 90% (TonoPEN AVIA) were discarded and the measurements were then repeated. The TonoLab, TonoVet, and TonoPEN AVIA had a detection limit of 7–60 mmHg, 10–60 mmHg, and 5–55 mmHg, respectively. Measurements were performed at baseline, 4 h, 8 h, 24 h, 4 days, 7 days, 14 days, 21 days, and 28 days. N equals the number of animals times the number of time points.

Reproducibility (also known as interobserver reproducibility) was defined as the ability to produce the same results for IOP measurements of identical samples under the same conditions by two different observers. Agreement (also known as intraobserver reproducibility) was defined as the ability of one observer to produce the same results of IOP in identical samples using different tonometers.

### Effect of sedation on IOP

IOP was measured using the TonoVet tonometer before (awake) and after sedation. The TonoVet was selected based on the results obtained in paragraph 2.2. First, the IOP of the left eye was measured sixfold. Rabbits were then IM sedated with medetomidine (0.5 mg/kg). Within 15 min after induction of sedation, the IOP of the left eye was measured again sixfold. Measurements were performed 1, 5, 7, 11, 15, 25, and 40 days after acclimatization.

### Sample size and statistical analysis

Sample size was determined using Meads resource equation^92^. IOP measurements were performed as part of another study^93^, hence the deviation in animal groups. All observed data were paired data between the two observers. Values were presented as mean IOP ± standard deviation (SD) for observer 1, observer 2, and both. To examine repeatability, IOP measurements were evaluated by coefficient of variation (CoV) as a normalized SD, as shown in;

$$CoV = \frac{SD}{{Mean}}x 100 (\% )$$

A smaller CoV means better repeatability. A CoV < 10% was indicative of good repeatability and a CoV < 5% of very high repeatability^94^. The CoV was calculated per measurement with the average providing a mean CoV.

Reproducibility was visualized by plotting mean values of observer 1 over observer 2 and calculating a linear regression line with 95% confidence interval (CI). Pearson’s correlation analysis was applied between both observers, followed by Bland and Altman analysis^95,96^. The Bland and Altman analysis compares the difference of the measurements versus the mean. Agreement of the different tonometers was also visualized through this method.

In calculating the influence of sedation, a two-way repeated measures ANOVA test was performed with Bonferroni correction for multiple testing to compare sedated to awake situations. In addition, the repeatability of measurements in sedated animals were plotted as Bland and Altman plots, including the difference between the measurement and mean along with the percentage of equality (agreement, lower is better) between the values and mean.

Tests were performed using GraphPad Prism version 9 (GraphPad Software inc., San Diego, CA, USA).

## Results

### Repeatability

An overview of the IOP results for all three tonometers, performed by both observers, is shown in Table 2. Mean IOP measured by the TonoLab was approximately three times higher for both observers compared to the TonoVet and TonoPEN AVIA. Both observers showed good repeatability (CoV < 10%) by using the TonoLab and TonoVet, while TonoPEN AVIA use resulted in poorer repeatability.

**Table 2:** Summary of IOP results of both observers. Measurements performed in triplicate, IOP expressed in mmHg.

	TonoLab	TonoVet	TonoPEN AVIA
**Observer 1**			
n	166	94	164
Mean IOP (SD)	37.00 (14.84)	11.41 (3.98)	11.76 (3.73)
Median IOP (25% PCTL − 75% PCTL)	34.00 (26.92–44.25)	11.00 (9.00–12.75)	11.67 (10.00–13.33)
CoV%	6.55	6.38	10.88
**Observer 2**			
n	166	94	164
Mean IOP (SD)	37.00 (14.24)	11.38 (3.71)	13.24 (5.78)
Median IOP (25% PCTL − 75% PCTL)	34.83 (27.92–44.00)	10.67 (9.33–13.08)	12.00 (10.00–14.25)
CoV%	7.04	7.08	14.78

Coefficient of variation (CoV), standard deviation (SD), percentile (PCTL).

**Figure 2:**
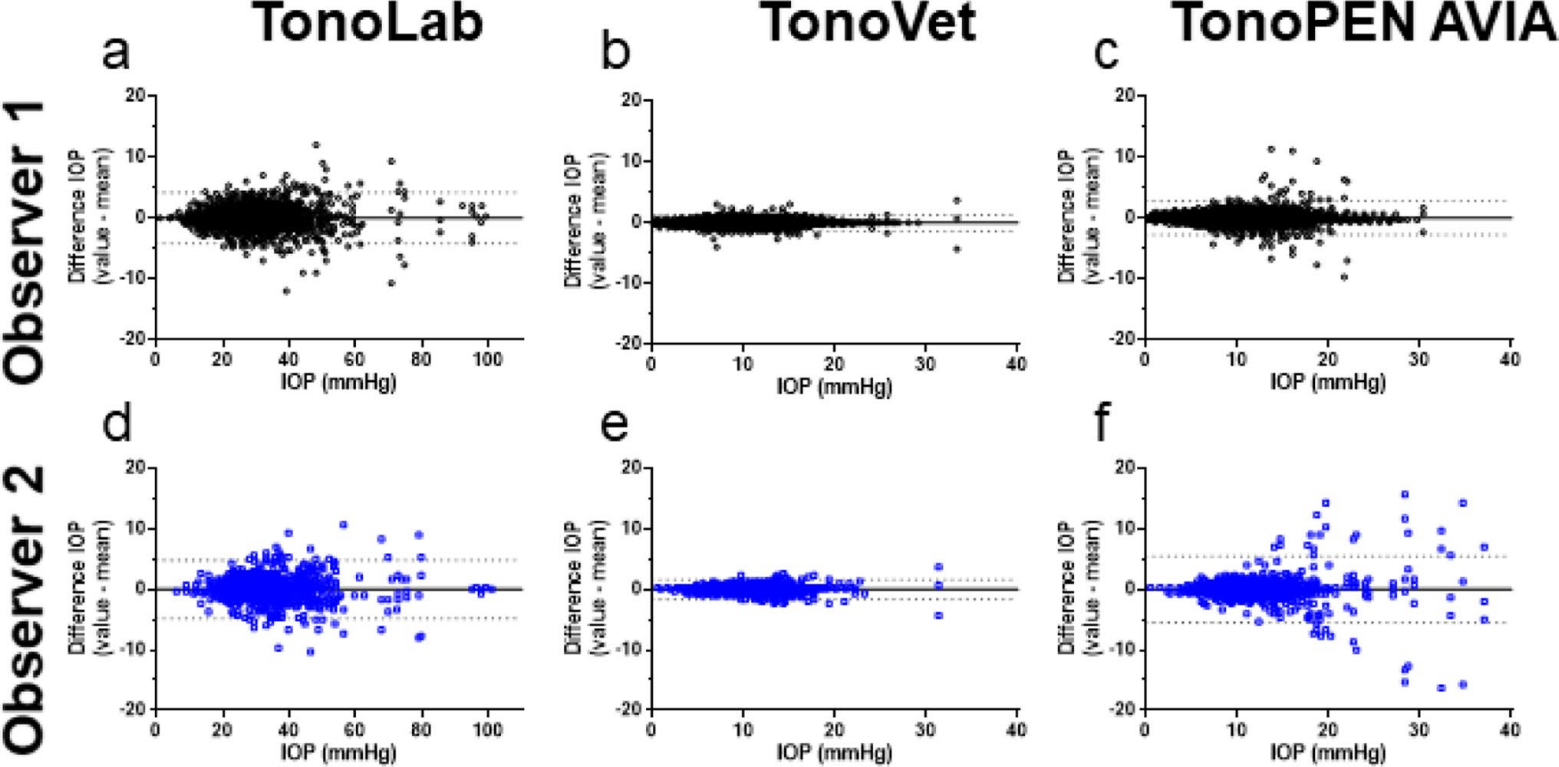
Repeatability of IOP measurements in tonometers (TonoLab (**a**,**d**), TonoVet (**b**,**e**), and TonoPEN AVIA (**c**,**f**)) for both observers. Difference per measurement (y-axis) is plotted over the mean IOP (x-axis). Dotted lines represent 1.96 times the SD (95% confidence interval).

The repeatability of IOP measurements for each tonometer and for each observer was visualized in a Bland and Altman plot, showing the difference between the individual values and the mean for repeated measurements (Fig. 2). The smallest deviation was observed for the TonoVet by both observers (1.34 and 1.65 for observer 1 and observer 2, respectively, Fig. 2b,e, dashed lines). The TonoLab showed a deviation of 4.17 and 4.81 for observer 1 and observer 2, respectively (Fig. 2a,d). Observer 1 showed a lower deviation than observer 2 when using the TonoPEN AVIA (2.76 vs 5.50) (Fig. 2c,f). This higher deviation of observer 2 could be caused by the increased scattering in higher IOP values.

**Figure 3:**
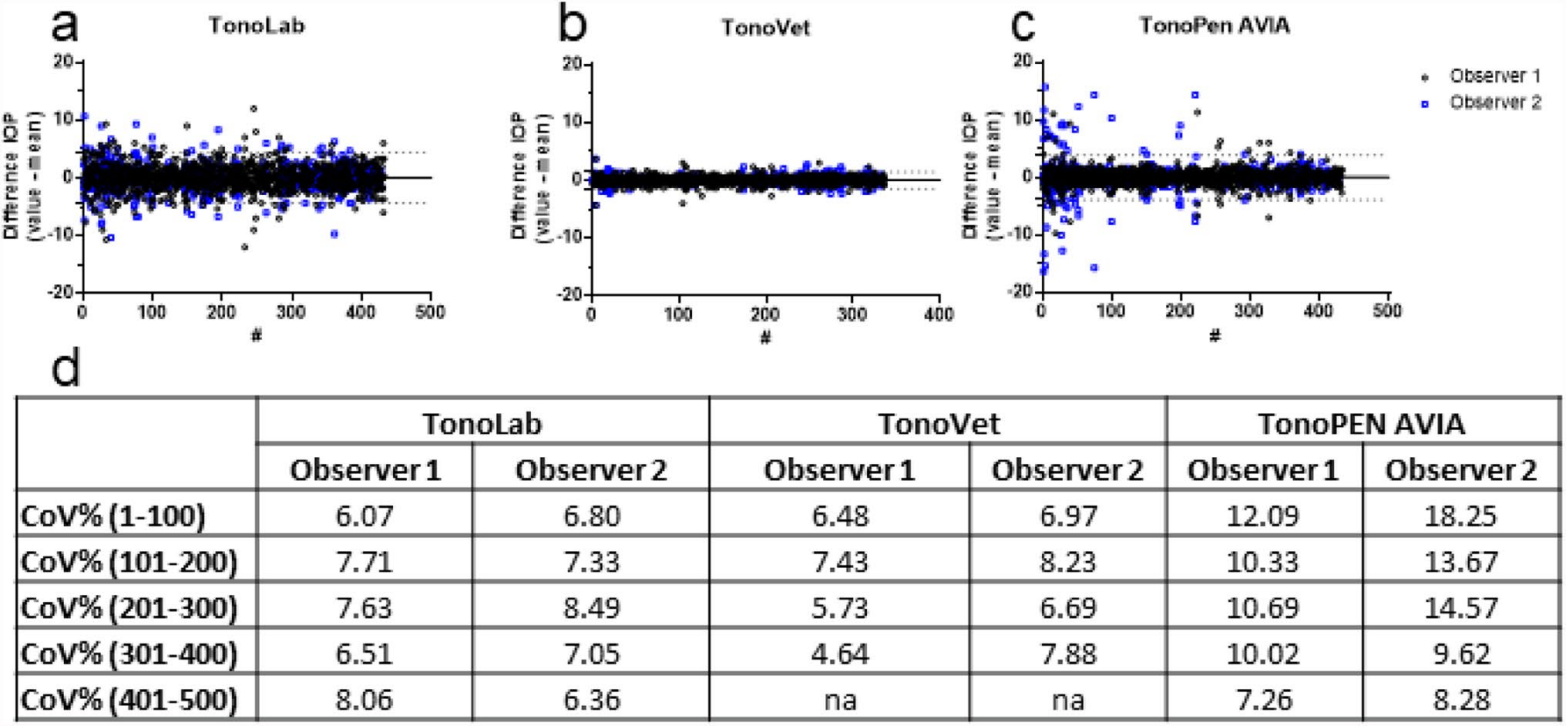
Repeatability of IOP measurements plotted over time. Intra-observer difference in IOP for the (**a**) TonoLab, (**b**) TonoVet, and (**c**) TonoPEN AVIA. (**a**–**c**) Dotted lines represent 1.96 times the SD (95% confidence interval (CI)). (**d**) Table summarizing the coefficient of variation (CoV) per range of measurements (per 100). Measurement #1 is the first measurement taken with the device, whereas #400 is the 400th measurement. na; not applicable.

When plotted over time (Fig. 3a,d), the repeatability of the TonoLab remained stable for both observers. For the TonoVet (Fig. 3b,d), the repeatability of observer 1 was improved over time (measurement 1–300 showed good repeatability (CoV < 10%) while measurement 301–400 showed very high repeatability (CoV < 5%)).This trend was similar for observer 2. Furthermore, during the earlier IOP measurements with the TonoPEN AVIA, more values were outside the 95% confidence interval (limit of agreement) for observer 2 (see Fig. 3c, and this correlated with the higher CoV% for measurement 1–100 (see Fig. 3d)). In addition, CoV% of IOP measurements from observer 2 decreased from 18.25% (measurement 1–100) to 8.28% (measurement 401–500) (Fig. 3d) indicating a learning curve when using the TonoPEN AVIA (observable by the funnel structure in Fig. 3c).

### Reproducibility

**Figure 4:**
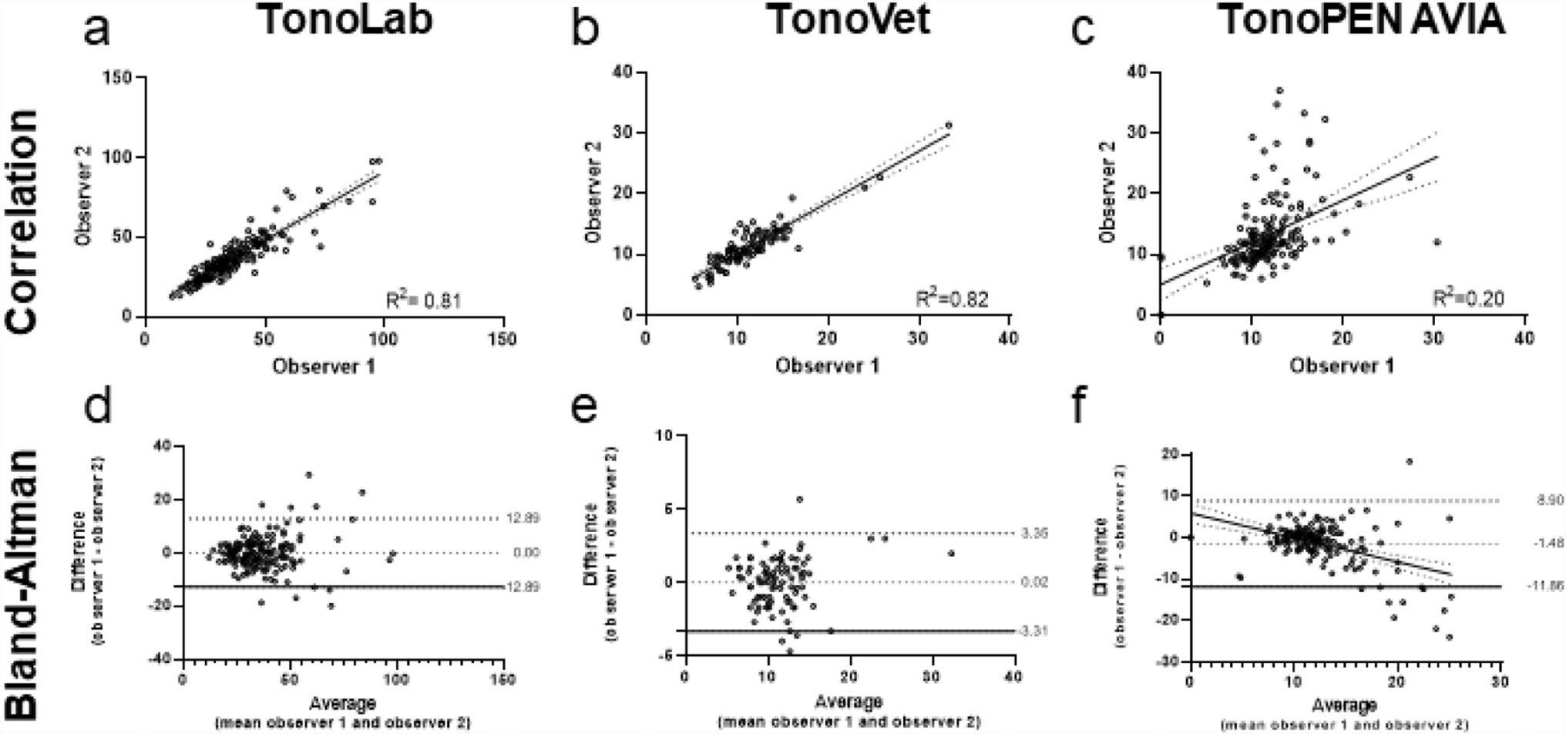
Reproducibility of different tonometers. (**a**–**c**) Show scatter plots with linear regressions (dashed lines is the 95% confidence interval (CI)). (**d**–**f**) Show a Bland and Altman plot expressing the difference of measurements by the observers plotted over the mean of the observers.

Figure 4 shows the reproducibility of the different tonometers of each observer. The TonoLab and TonoVet displayed a very strong correlation between observers (r = 0.90, *p* < 0.0001, R^2^ = 0.8, for both Fig. 4a,b, respectively), while the TonoPEN AVIA showed a moderate correlation (r = 0.45, *p* < 0.0001, R^2^ = 0.2 (Fig. 4c)).

Both observers obtained similar results with the TonoLab (a bias of 0.0 with a deviation of ± 12.89 (Fig. 4d)). IOP values measured by observer 2 were on average 0.2 mmHg lower than those measured by observer 1 using the TonoVet, (a bias of 0.2 with a deviation of ± 3.3 (Fig. 4e)). Furthermore, IOP values measured by observer 1 were on average 1.48 mmHg lower than those measured by observer 2 with the TonoPEN AVIA (bias of − 1.48 with a deviation of ± 10.3 (Fig. 4f)). The negative bias was mainly caused by the large difference between both observers in the higher IOP values, as shown by the linear regression line (R^2^ = 0.20) in the plot.

### Agreement between different tonometers

**Figure 5:**
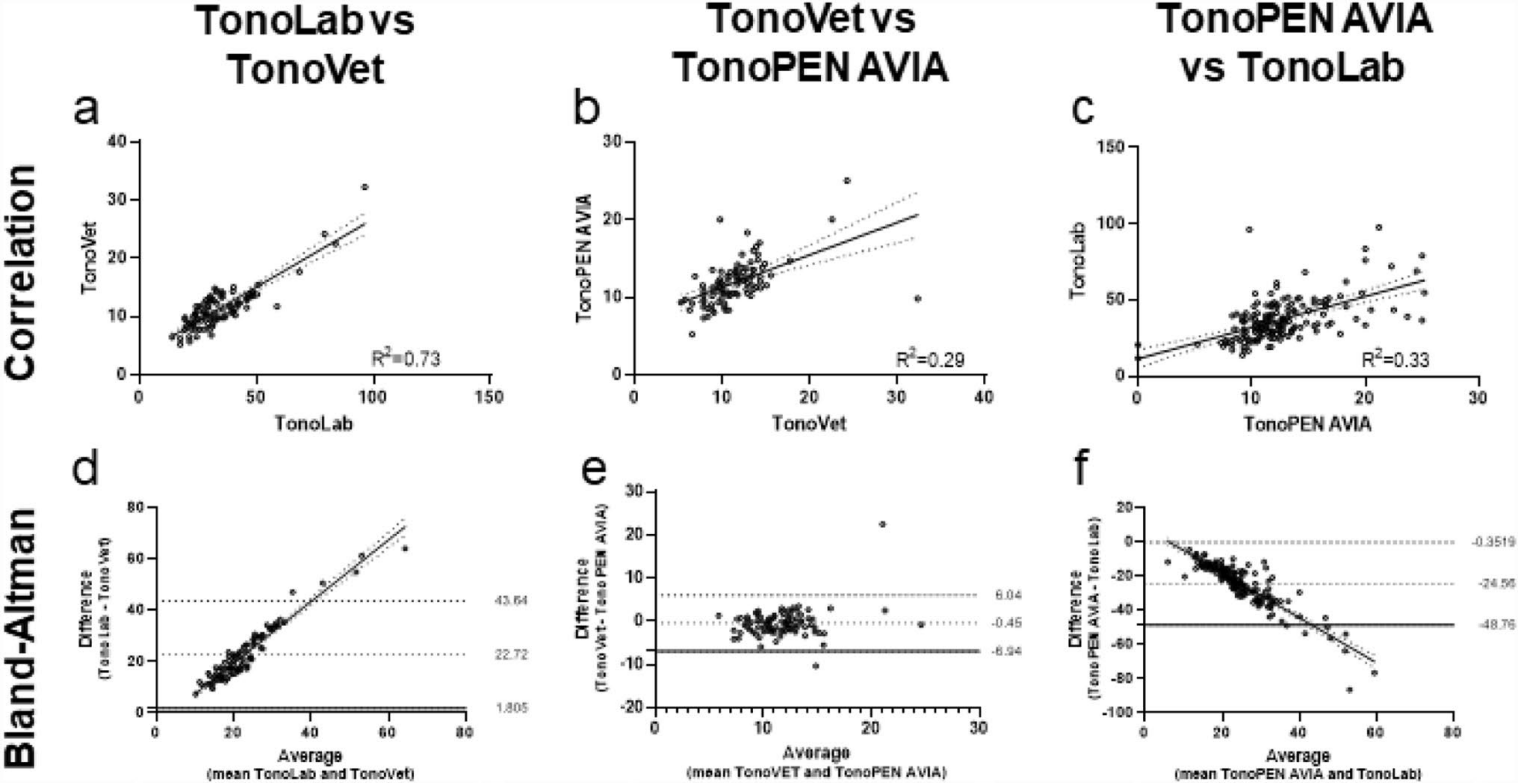
Agreement between tonometers. (**a**–**c**) Show a scatter plot with linear regression (dashed lines is the 95% confidence interval (CI)). (**d**–**f**) Show a Bland and Altman plot expressing the difference of measurements by the tonometers plotted over the mean values of the tonometers.

Agreement between the different tonometers was assessed by combining data of observer 1 and 2 (Fig. 5). Data for the individual observers is shown in Fig. S1. A strong correlation was observed between the TonoLab and TonoVet (r = 0.85, *p* < 0.0001, R^2^ = 0.73, Fig. 5a). A moderate correlation was found for the comparison of the TonoPEN AVIA with the TonoVet, and the TonoLab with TonoPEN AVIA (r = 0.53, *p* < 0.0001, R^2^ = 0.29, Fig. 5d and r = 0.58, *p* < 0.0001, R^2^ = 0.33, Fig. 5c, respectively). Due to the learning curve of observer 2 for the TonoPEN AVIA, a lower correlation and linear regression were obtained when compared to observer 1 (Fig. S1b, c, e, and f). A moderate correlation was found for the comparison of the TonoPEN AVIA with the TonoVet for observer 1, whereas this was low for observer 2 (r = 0.60, *p* < 0.0001, R^2^ = 0.36, Fig. S1b and r = 0.35, *p* < 0.0001, R^2^ = 0.12, Fig. S1e, respectively). A similar pattern was observed when comparing the TonoLab with TonoPEN AVIA for observer 1 and observer 2 (r = 0.63, *p* < 0.0001, R^2^ = 0.40, Fig. S1d and r = 0.40, *p* < 0.0001, R^2^ = 0.16, Fig. S1f, respectively).

After plotting the difference between the tonometers, the TonoLab showed on average 22.7 ± 20.9 mmHg higher IOP values than the TonoVet (Fig. 5d). This difference was caused by the higher absolute IOP values of the TonoLab compared to the TonoVet, additionally confirmed by the linear regression line (R^2^ = 0.92).

The TonoVet and TonoPEN AVIA were more in agreement with a bias of 0.45 (the TonoVet provided a slightly higher IOP value than the TonoPEN AVIA) with a deviation of ± 6.5 (Fig. 5e). The TonoLab and TonoPEN AVIA showed a negative trend when differences were plotted against the mean values (R^2^ = 0.80, Fig. 5f). Similar to the comparison with the TonoVet, a bias of − 24.6 ± 24.2 mmHg was observed between the TonoLab and the TonoPEN AVIA, due to the high absolute values measured by the TonoLab. The findings were in line with individual differences between tonometers (Fig. S1g-l).

### Effect of sedation on IOP

**Figure 6:**
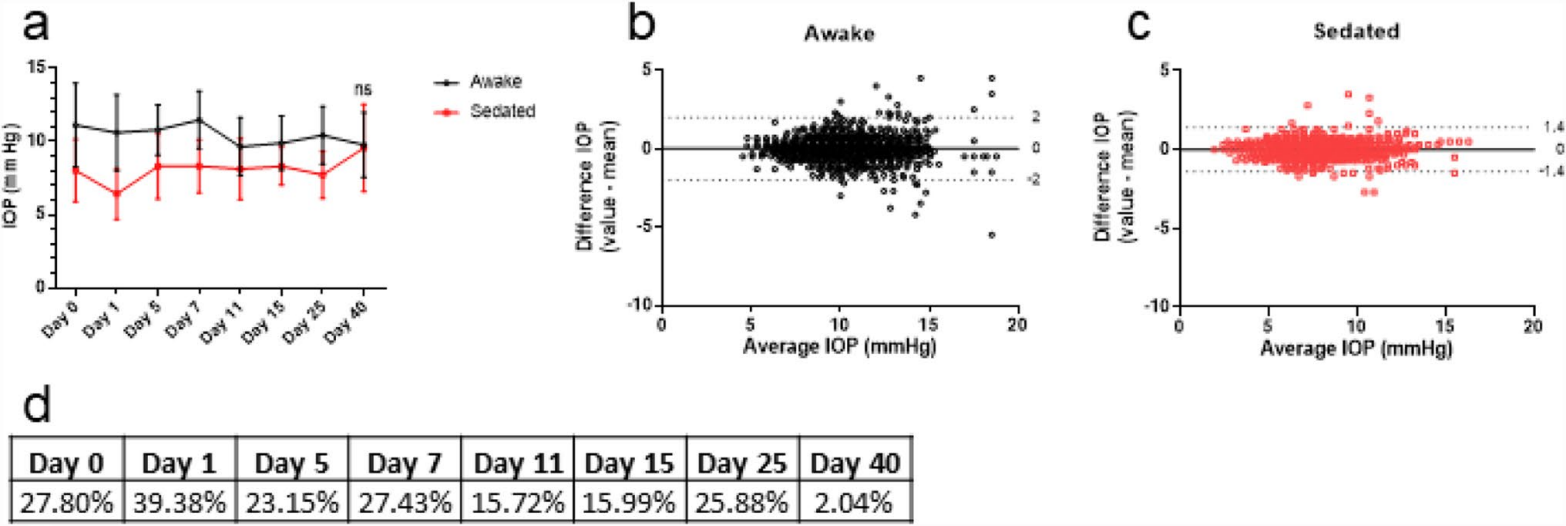
Effect of sedation on IOP. Measurements taken by the same observer using the TonoVet. (**a**) IOP over time in both awake and sedated rabbits. (**d**) Percentage difference in IOP between awake and sedated rabbits. (**b**,**c**) Bland and Altman plot from the awake and sedated rabbits. Dotted lines show 1.96 times the SD (95% confidence interval). ns; not significant.

Figure 6a shows IOP over time in awake and sedated animals, measured with the TonoVet by observer 2. A two-way repeated measures ANOVA showed a significant difference between awake and sedated IOP measurement on all days, (Table 3, Fig. 6a) except for day 40 (*p* > 0.9999). For day 0 until day 25, the IOP of sedated animals was about 25% lower than of awake animals, while on day 40 this difference was only about 2% (Fig. 6d). The repeatability of the measurement was not affected by sedation, as shown in the Bland and Altman plots (Fig. 6b,c).

**Table 3:** Results of the two-way repeated measures ANOVA with Bonferroni correction, N = 21.

	Mean awake	Mean sedated	Mean diff	SE of diff	95% CI of diff	P Value
Day 0	11.13	8.03	3.09	0.45	1.80 to 4.39	**< 0.0001**
Day 1	10.61	6.43	4.18	0.31	3.31 to 5.04	**< 0.0001**
Day 5	10.80	8.30	2.50	0.34	1.55 to 3.45	**< 0.0001**
Day 7	11.48	8.33	3.15	0.40	2.04 to 4.26	**< 0.0001**
Day 11	9.65	8.13	1.52	0.37	0.48 to 2.55	**0.0007**
Day 15	9.90	8.32	1.58	0.29	0.77 to 2.40	**< 0.0001**
Day 25	10.43	7.73	2.70	0.33	1.78 to 3.62	**< 0.0001**
Day 40	9.78	9.58	0.20	0.48	− 1.14 to 1.54	> 0.9999

CI, confidence interval.

## Discussion

In this study, we calculated the reproducibility, repeatability and agreement of three different tonometers (the TonoLab, the TonoVet and the TonoPEN AVIA) in a cohort of normotensive NZW rabbits, alongside the effect of sedation on IOP measurements.

Our results showed higher absolute IOP values when using the TonoLab compared to the TonoVet and the TonoPEN AVIA. Since the TonoLab is designed for use in mice and rats, the thicker cornea of the rabbit probably affected the readings of this device^97,98^. The TonoVet showed the highest repeatability (CoV of 6.38%), followed by the TonoLab and TonoPen AVIA (CoV of 6.55% and 10.88%, respectively). Furthermore, the repeatability was acceptable (defined as CoV < 10%)^94^ for the TonoVet and TonoLab and the measurements were in line with previous reports that found a CoV of 6.50% and 10.30% for the TonoVet and TonoPEN XL (an older version of the TonoPEN AVIA), respectively^53^.

Applanation tonometry is known to be sensitive to the technique used as well as the force applied^6^, while rebound tonometers can be easier to use. The ease of use for the TonoVet and TonoLab was comparable between the two, both allowing probes to be easily installed and correct usage of the device to be learned quickly. However, the TonoPEN AVIA showed to have a steeper learning curve. Applying the tip-cover of the TonoPEN AVIA may also introduce additional errors. Results showed that (for the TonoPEN AVIA in particular) extremely high IOP values (above 20 mmHg) are prone to larger error, in line with previous studies^51,54^. The highest correlation with regard to reproducibility was found with the two rebound tonometers, TonoLab and TonoVet, with a lower reproducibility of the TonoPEN AVIA. A possible explanation for this might be that using the TonoPEN AVIA is more difficult.

On agreement between the different tonometers, the TonoLab and TonoVet showed a strong correlation (r = 0.85, R^2^ = 0.73, *p* < 0.0001), in line with our expectations as both measure the IOP via rebound tonometry. When comparing the TonoPEN AVIA with the TonoVet, and the TonoLab with the TonoPEN AVIA, a more moderate agreement correlation was found (r = 0.53, *p* < 0.0001, and r = 0.58, *p* < 0.0001, respectively). Pereira et al*.* showed a correlation of r = 0.60 (R^2^ = 0.36) between the TonoVet and the TonoPEN AVIA in a cohort of 76 rabbit eyes^51^, in line with our findings. Ma et al*.* compared the TonoVet to the TonoPEN XL in rabbits. They found a high linear regression (R^2^ = 0.98) between both tonometers, but correlation was not tested separately^53^.

Recently, Gloe et al*.* examined the TonoVet Plus (a novel version of the TonoVet that has a rabbit setting, released after the onset of this study), TonoVet, TonoPEN Vet, and TonoPEN AVIA on *post-mortem* rabbit eyes. Their results showed a high linear regression of the tonometers when compared to manometry, the TonoVet Plus (R^2^ = 0.99), the TonoVet (R^2^ = 0.98), the TonoPEN Vet (R^2^ = 0.92), and the TonoPEN AVIA (R^2^ = 0.92). However, no correlation between the tonometers was done. Their findings indicate that all tonometers tend to underestimate IOP when compared to manometry^54^.

In the present study, the TonoVet and TonoPEN AVIA showed the highest agreement; however, the correlation is moderate due to the different working mechanisms of the tonometers (rebound versus applanation). The TonoLab and TonoVet demonstrated the best correlation, however their agreement was lower because the measurements of the TonoLab showed a much higher IOP than the TonoVet. Since the probe size of the TonoLab is specifically designed for use in small rodents, the system is not calibrated for the thick corneas of rabbits^35^. The average central corneal thickness (CCT) has been reported to be 105 µm for mice and 130 µm for rats^35^. The average CCT of New Zealand White rabbits is 407 ± 20 µm^99^, 3 to 4 times thicker than the reported values in rodents. The rabbit’s greater corneal thickness is likely responsible for inaccurate IOP values obtained with the TonoLab. Although we did not measure CCT in every animal and could therefore not correct for CCT differences, they were all New Zealand White rabbits from the same age, and differences in CCT were therefore likely to be limited . Additionally, all animals were measured with all three devices and the aim was to observe the differences between those devices .

Overall, the TonoVet was the best tonometer in terms of repeatability, reproducibility, and agreement and it was the most consistent tonometer in comparison to the TonoPEN AVIA and TonoLab. The TonoVet showed the highest agreement and strongest correlation to the other tonometers. The correlation between the TonoLab and TonoPEN AVIA was found to be moderate, presumably from them being different mechanisms of measurement, similar to the TonoPEN AVIA and the TonoVet having a moderate correlation.

The effect of the selective alpha 2-adrenoceptor agonist medetomidine on IOP was examined using the TonoVet. Our results indicated a ~ 25% reduction in IOP after IM sedation. In dogs, no reduction of IOP has been observed using a similar dose of 0.5 mg/kg medetomidine IM^100^. In rabbits, medetomidine has also topically been instilled^18,19^. Two studies found that a dose of 25 μg medetomidine reduced the IOP of the contralateral eye in 30 min by ~ 50%, while the treated eye was not affected^18,19^. This effect has also been observed in dogs^101^.

In contrast to other time points in our study, no difference in IOP between awake and sedated animals was observed at day 40. This might have been caused by elevated mental stress levels in the rabbits, caused by euthanasia of animals performed in the same room, an effect also observed in dogs^101^. Levels of mental stress were not assessed during the study but did not affect the repeatability and reproducibility of the measurements.

Because IOP fluctuates over short periods of time, similar to fluctuations in heart rate during the day^15^, any tonometer that records a near-instantaneous measurement of IOP is taking a sample from the IOP cycle causing measurements to only provide an estimation of the IOP at one time point. Variables such as fluctuating blood pressure, pulse, respiration, and anxiety could also account for discrepancies in IOP, along with the mental stress of repeated measures^102^.

## Conclusion

Of the three tonometers tested, TonoVet was the most favorable as it showed the smallest inter- and intra-observer variations, without a learning curve. The TonoLab showed three-fold higher IOP values compared to the TonoVet, making it unsuitable for determining rabbit IOP. Additionally, when IM sedation is required in future experiments, it should be taken into account that it significantly reduces the IOP of rabbits (often by more than 25%).

## Supplementary Information

Supplementary Figure S1.

## Abbreviations

ARRIVE: Animal Research: Reporting of In Vivo Experiments
ARVO: Association for Research in Vision and Ophthalmology
AH: Aqueous humor
CI: Confidence Interval
CCT: Central corneal thickness
CoV: Coefficient of variation
GAT: Goldmann applanation tonometry
IM: Intramuscular
IOP: Intraocular pressure
NZW: New Zealand White
ns: Not significant
SD: Standard deviation
