# Supplementary material for: Repeatability, reproducibility, and agreement of three tonometers for measuring intraocular pressure in rabbits
Source: Sci Rep. 2021 Sep 28;11:19217. doi: 10.1038/s41598-021-98762-7 (PMC8478901; doi:10.1038/s41598-021-98762-7)
Supplement: Supplementary file 1 — Supplementary Figure S1. [file 41598_2021_98762_MOESM1_ESM.docx]

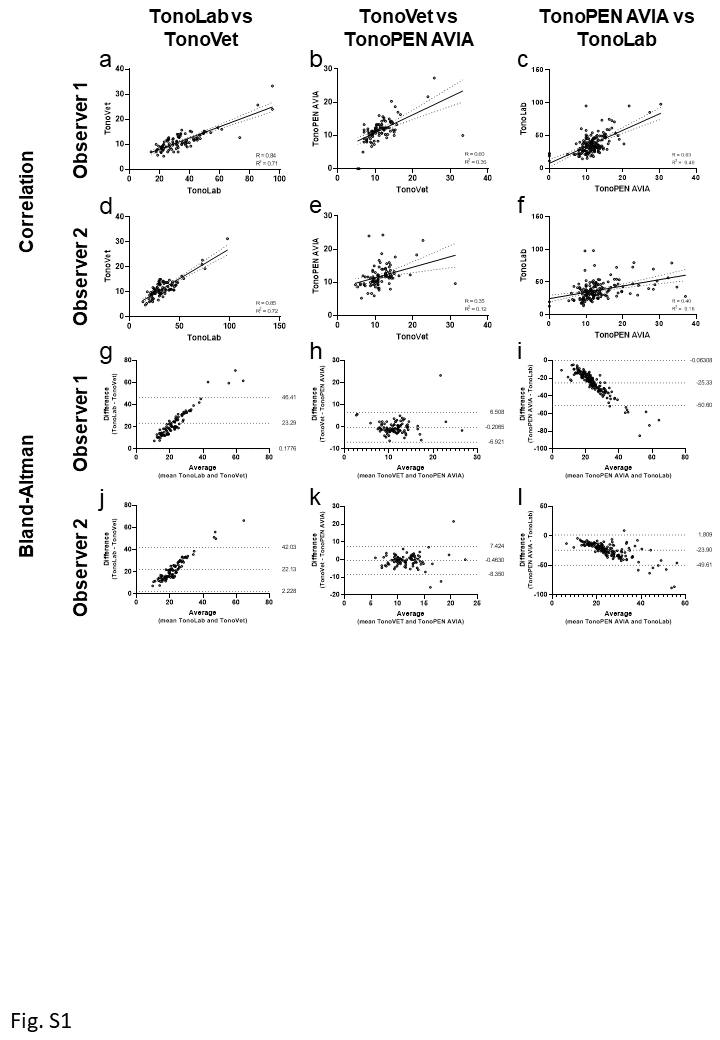


*Figure S1. Agreement between different tonometers per observer. A-f, show a scatter plot with linear regression (dashed lines is the 95% confidence interval (CI)). G-l, show a Bland and Altman plot expressing the difference of measurements by the tonometers plotted versus the mean-values of the tonometers.*
